# Evaluating the Transition from 3D Laparoscopy to Robotic Partial Nephrectomy: Trifecta Achievement and Nephrometry Score Differences

**DOI:** 10.3390/cancers17243976

**Published:** 2025-12-13

**Authors:** Piotr Kania, Paweł Marczuk, Jakub Biedrzycki, Markijan Kubis, Szymon Kania, Kajetan Juszczak, Maciej Salagierski

**Affiliations:** 1 Department of Urology and Urological Oncology, St. John Paul II Masovian Regional Hospital, Ks. Jozefa Poniatowskiego 26, 08-110 Siedlce, Poland; 2 Faculty of Medicine, University of Siedlce, 08-110 Siedlce, Poland; 3 Urology Department, Collegium Medicum, University of Zielona Góra, 65-417 Zielona Góra, Poland; 4 Medical University of Lublin, 20-059 Lublin, Poland; 5 Department of Urology and Andrology, Collegium Medicum, Nicolaus Copernicus University, 85-067 Bydgoszcz, Poland

**Keywords:** robot-assisted partial nephrectomy, three-dimensional laparoscopy, complete transition, nephron-sparing surgery, TRIFECTA, RENAL nephrometry score, warm ischemia time

## Abstract

Background: Partial nephrectomy (PN) is the standard treatment for localized renal tumors where nephron preservation is feasible. The evolution of minimally invasive surgery has progressed from conventional two-dimensional laparoscopy to three-dimensional (3D) laparoscopy and, more recently, to robotic-assisted techniques. Although robotic and laparoscopic PN have been widely compared, evidence focusing on a complete transition from 3D laparoscopy to robot-assisted partial nephrectomy (RAPN) remains scarce. Methods: This retrospective single-surgeon study included 80 consecutive patients treated between 2018 and 2024, encompassing the full transition period from 3D LPN to RAPN. Thirty-six patients underwent 3D laparoscopy and forty-four underwent robotic surgery, excluding the first ten robotic cases representing the learning phase. Propensity score weighting was applied to minimize baseline differences. Results: Tumors treated with RAPN had significantly higher RENAL scores (median 8 vs. 6, *p* = 0.001), indicating greater complexity, while perioperative outcomes—including hospital stay, operative time, and complication rates—remained comparable. Warm ischemia time was significantly shorter in the RAPN group (17.5 vs. 22 min, *p* = 0.005), and the TRIFECTA rate was higher though not statistically significant. Conclusions: These results indicate that a complete transition from 3D laparoscopy to robotic partial nephrectomy is safe and feasible, maintaining or improving outcomes even in more complex tumors and broadening the applicability of nephron-sparing surgery.

## Simple Summary

Minimally invasive partial nephrectomy represents the treatment of choice for localized renal tumors, provided that complete tumor excision with preservation of renal function is technically achievable. Over the past decade, surgical technology has evolved from conventional two-dimensional laparoscopy to three-dimensional (3D) laparoscopy and, more recently, to robotic-assisted surgery. Although many studies have compared robotic and laparoscopic partial nephrectomy, most were based on older 2D laparoscopy. Our study presents the experience of a complete transition from 3D laparoscopic to robotic partial nephrectomy performed by a single surgeon in a specialized center. Despite operating on significantly more complex tumors in the robotic group, perioperative outcomes—including complication rate, hospital stay, and renal function preservation—were comparable or better than in 3D laparoscopy. These results show that for surgeons already proficient in 3D laparoscopy, switching to robotic surgery can be safe and efficient, offering advantages, particularly in anatomically complex kidney tumors. The robotic system extends the possibilities of nephron-sparing surgery and may help to reduce the rate of radical nephrectomies.

## 1. Introduction

Renal cell carcinoma ranks 14th on the list of cancers, representing around 2% of all cancers [1,2]. Organ-confined renal cancer (RC) is primarily treated through surgical resection. Nephron-sparing surgery (NSS) remains the gold standard treatment for localized renal cancer where technically feasible. The advantage of partial nephrectomy (PN) lies in preservation of parenchyma and hence renal function. However, this advantage is counterbalanced with increased surgical risk [3,4]. Minimally invasive surgery (MIS) in comparison with open surgery offers a number of benefits, including smaller incisions, less morbidity, faster recovery, reduced pain, shorter length of hospital stay, and improved cosmesis [5]. PN for kidney tumors up to 7 cm in diameter (clinical staging up to T1b) has shown benefits in preserving renal function and potentially reducing cardiovascular mortality rates compared to radical nephrectomy (RN) [6]. Nevertheless, both surgical methods yield similar oncological outcomes [7]. Since 1993, laparoscopic approaches for PN have been developed, providing the advantages of MIS [8]. PN was considered as one of the most demanding procedures in laparoscopic urology as it requires high skills and confidence in fast and precise tumor resection as well as skills in intracorporeal laparoscopic suturing. Due to its complexity and steep learning curve, it is limited to highly specialized centers. By integrating three-dimensional endoscopes into laparoscopic practice, some institutions have been able to reduce the challenges posed by two-dimensional, plain visual limitations [9], thus approaching the view seen by the surgeon through the robotic console. Evidence from meta-analyses suggests that prior laparoscopic training enhances surgeons’ ability to perform procedures in the robotic setting [10]. Available evidence indicates that proficiency in three-dimensional laparoscopy for partial nephrectomy may be achieved earlier, which could facilitate a smoother transition to robotic-assisted partial nephrectomy [11]. Further development of MIS in localized renal cancer led to robot-assisted surgery (RAS), and this approach has broadened the use of MIS in PN. Robot-assisted laparoscopy through improved precision of preparation, higher degree of freedom of the instruments and high definition, three-dimensional (3D) imaging overcomes classic laparoscopy limitations like, for example, steep learning curve and prolonged operative time [12]. Despite the well-documented benefits of robotic-assisted partial nephrectomy (RAPN) for both patients and surgeons, robotic-assisted surgery (RAS) remains less accessible in some regions due to economic and healthcare system limitations. Consequently, some centers continue to use traditional laparoscopy for PN to offer the advantages of MIS. Surgery assisted by robotic platforms has been under development for nearly thirty years. However, in some regions, the transition from traditional laparoscopy to robot-assisted surgery in nephron-sparing surgery (NSS) is still forthcoming. Therefore, the purpose of the study was to compare TRIFECTA rate, RENAL nephrometry score, and postoperative outcomes between 3D laparoscopic PN and robot-assisted PN during the transition period from laparoscopic to robotic PN.

## 2. Material and Methods

***Study design and populations.*** This retrospective cohort study included 80 consecutive patients who underwent NSS for renal tumors in one institution between 2018 and 2024. This period encompassed the transition from three-dimensional (3D) laparoscopy to robotic techniques. In years 2018–2021, thirty-six laparoscopic nephron-sparing surgeries were performed, while in years 2021–2024 fifty-four robot-assisted partial nephrectomies were performed. All patients with renal tumors were eligible for NSS or radical nephrectomy based on technical feasibility. Surgeries were conducted by an experienced robotic surgeon, who has performed several hundred laparoscopy procedures and has completed the learning curve for robot-assisted surgeries, primarily radical prostatectomies. Patients were divided into two groups: (1) laparoscopic partial nephrectomy (LPN, *N* = 36) and (2) robot-assisted partial nephrectomy (RAPN, *N* = 44). The first ten patients operated with robotic assistance were excluded from the evaluation as a learning curve. This threshold was chosen according to reports from early robotic programs, which identified significant variability in perioperative outcomes during the initial learning phase of approximately 10–15 RAPN cases [13]. The study was approved by the Institutional Review Board (approval number: 10/2024). All patients received detailed information about the available surgical options, including nephron-sparing surgery and radical nephrectomy, as well as the possible surgical approaches (laparoscopic or robot-assisted). Written informed consent was obtained from each patient prior to surgery.

We present a retrospective analysis of prospectively collected data of partial nephrectomy (PN) performed using 3D laparoscopy, involving a cohort of patients who underwent robotic-assisted PN following the introduction of a robotic system. All procedures were performed in a single hospital unit by the same surgeon, following a complete transition from three-dimensional laparoscopy to robotic-assisted surgery.

***Trifecta and RENAL score.*** TRIFECTA was defined as the composite outcome of negative surgical margins (R0 status), absence of perioperative complications (Clavien-Dindo grade III or higher) and preservation of renal function (defined as a warm ischemia time under 25 min and maintaining over 90% of estimated glomerular filtration rate). Moreover, in this study, the relationship between the preoperative RENAL nephrometry score and the surgical decision between radical and partial nephrectomy in both groups was analyzed. RENAL score assesses the complexity of the tumor, based on tumor size, exophytic/endophytic proportions, proximity to the collecting system or renal sinus and location relative to the polar lines. Every tumor can receive between 4 and 12 points. Low complexity is defined as 4–6 points, medium complexity as 7–9 points, and tumors that were assessed for at least 10 points are considered as complex lesion. This analysis aimed to determine whether patients with higher RENAL scores, reflecting greater tumor complexity, were more likely to undergo PN via robot-assisted technique compared to 3D laparoscopic PN (LPN).

***Postoperative outcomes.*** The postoperative outcomes between 3D laparoscopic PN and RAPN were assessed as follows: (1) the length of hospital stay (LOS), (2) 30-day morbidity, (3) postoperative complications, and (4) intraoperative factors such as operation time, warm ischemia time (WIT), and the necessity of using double layer renorrhaphy. LOS was measured in days from surgery to discharge. Thirty-day morbidity included any adverse event within thirty days post-surgery. Postoperative complications were classified using the Clavien–Dindo system, with grades I–II considered minor (e.g., wound infection requiring antibiotics) and grades III–V considered major (e.g., reoperation, organ failure). Operation time was measured from preparation of surgical field to wound closure. Warm ischemia time (WIT) was defined as the duration of renal artery clamping during the procedure. The requirement for both internal and external renal parenchymal closure in securing adequate hemostasis was also analyzed. These intraoperative variables were examined to elucidate potential disparities in surgical efficiency and technical demands associated with each approach.

***Surgical technique.*** The same technique was employed for both 3D laparoscopic and robotic PN The patient was placed at a 90-degree flank position, with the table slightly bent at the waist to widen the space between the iliac crest and costal margin. The patient was secured with multiple broad adhesive strips and carefully padded. The four-trocar method was used for left-side tumors in LPN, with an additional right-side trocar to retract the liver. For the robot-assisted approach, an extra 12 mm trocar was required for the assistant. Intraoperative ultrasound was used in all cases to help localize the lesion, assess tumor boundaries, and evaluate renal vasculature, particularly in the presence of perinephric toxic fat. Recent evidence highlights its pivotal role in improving surgical precision during 3D laparoscopic partial nephrectomy [14]. Dissection of renal hilum was aimed to place a secure vascular tape over a major artery. All procedures (both 3D laparoscopic and robotic PN) were performed using on-clamp technique. Following the clamping of the renal artery using a Bulldog clamp, (Dufner, Germany) the tumor was excised along with a margin of healthy parenchyma. Internal renorrhaphy was meticulously performed using barbed 2.0 sutures, and the defect was covered with a fibrin sealant patch in most cases. After renal artery unclamping hemostasis was assessed. If hemostasis was inadequate, the following measures were implemented: tightening of the internal renorrhaphy, external renorrhaphy placement with interrupted sutures blocked by polymeric clips, and placement of a bolster of oxidized cellulose beneath these sutures. The patient was discharged upon the return of bowel function and the ability to tolerate a regular diet.

***Statistical analysis.*** Statistical analyses were performed with a predetermined significance level of *p*-value ≤ 0.05. The normality of continuous variable distributions was evaluated through application of the Shapiro–Wilk test. For continuous variables exhibiting departures from normality, descriptive statistics were presented as medians accompanied by interquartile ranges (*IQRs*), delineated by the first (*Q1*) and third (*Q3*) quartiles. Categorical variables were characterized using absolute frequencies (*n*) and relative proportions expressed as percentages (%). To examine differences in numerical variables across two independent groups, the Wilcoxon rank-sum test was utilized for data not adhering to a normal distribution, ensuring a non-parametric approach suitable for skewed distributions. Associations between categorical variables were investigated using Pearson’s chi-square test; in instances where expected cell frequencies were insufficient, Fisher’s exact test was applied. To address the primary objective, propensity score weighting (PSW) was employed to balance baseline confounders between the laparoscopy and robotic groups, ensuring comparability and reducing selection bias. PSW included following covariates as predictors: sex, age, BMI, ASA score, previous surgery, baseline creatinine, baseline eGFR, and RENAL score.

Missing data (e.g., RENAL score, *N* = 74; ASA score, *N* = 79) were handled using multiple imputations with chained equations, generating five imputed datasets under the assumption that the data were missing at random, in order to retain the full sample (*N* = 80) for analysis. Weighted univariate regression analyses were subsequently performed to compare postoperative outcomes between the two groups. For binary outcomes, weighted logistic regression was used, and adjusted odds ratios (ORs) with 95% confidence intervals (CIs) were reported. For the continuous outcome, weighted linear regression was employed, and adjusted mean differences (MDs) with 95% CI were calculated. *p*-values were derived from these models, with a threshold of *p* < 0.05 considered statistically significant.

## 3. Results

### 3.1. Patient’s Characteristics

The current analysis presents a comparative analysis of patient demographics, tumor characteristics, and surgical outcomes between DaVinci robotic-assisted partial nephrectomy (RAPN) and laparoscopic PN (LPN) for renal tumors in a cohort of 80 patients. Detailed patient’s characteristics and preoperative factors that may influence surgery outcomes are presented in Table 1. Cohorts in both groups were comparable in demographics, comorbidities and preoperative renal function.

***RENAL and TRIFECTA.*** The RENAL score in the RAPN group with median score of 8 points was statistically significant higher compared to the LPN group with median score of 6 points (*p*-value = 0.001). This is backed by the RENAL complexity category breakdown (*p* = 0.001): only 28.21% of RAPN tumors were low complexity versus 65.71% for LPN (*p* = 0.001), while medium complexity jumped to 61.54% in RAPN versus 34.29% in LPN (*p* = 0.073), and high complexity was exclusively RAPN at 10.26% versus 0% in LPN (*p* = 0.123). Tumor location also differed significantly (*p* = 0.020), with RAPN dominating posterior tumors at 48.72% versus 20.00% for LPN (*p* = 0.031) and lagging in central tumors at 23.08% versus 48.57% (*p* = 0.016). It is proven that transition from laparoscopic to robot-assisted partial nephrectomy allowed us to apply more complex renal tumors to minimally invasive nephron-sparing techniques. Comparison of primary outcomes, which includes differences in RENAL score and TRIFECTA rate, is presented in Table 2. The TRIFECTA rate was higher in the RAPN group, but difference between two cohorts was not statistically significant. Differences in components of TRIFECTA, such as resection margin status, major postoperative complications, and postoperative renal function were insignificant too. The warm ischemia time was significantly shorter in RAPN with median of 17.5 min when compared to LPN with median of 22 min (*p* = 0.005).

***Postoperative outcomes.*** The differences in length of hospital stay, 30-day morbidity, postoperative complications, the necessity of second suture layer (double renorraphy), and operation time did not differ significantly between RAPN and LPN. Warm ischemia time was significantly shorter in RAPN when compared to LPN (Table 2 and Table 3). Although lower WIT in the RAPN group suggested better postoperative renal function, the difference in eGFR was not statistically significant.

### 3.2. Propensity Score Weighting and Covariate Balance

The results delineated in Table 4 were derived from a univariate regression framework applied after propensity score weighting (PSW), a statistical technique utilized to attenuate confounding by equilibrating baseline covariates between the DaVinci robotic-assisted PN (RAPN) and laparoscopic PN (LPN) groups. Other outcomes, such as TRIFECTA rate, length of hospital stay, and postoperative complications were comparable between groups.

Cohorts in both groups were comparable in demographics, comorbidities, and preoperative renal function.

***RENAL and TRIFECTA.*** The RENAL score in the RAPN group with median score of 8 points was statistically significantly higher compared to the LPN group with median score of 6 points (*p*-value = 0.001). This is backed by the RENAL complexity category breakdown (*p* = 0.001): only 28.21% of RAPN tumors were low complexity versus 65.71% for LPN (*p* = 0.001), while medium complexity jumped to 61.54% in RAPN versus 34.29% in LPN (*p* = 0.073), and high complexity was exclusively RAPN at 10.26% versus 0% in LPN (*p* = 0.123). Tumor location also differed significantly (*p* = 0.020), with RAPN dominating posterior tumors at 48.72% versus 20.00% for LPN (*p* = 0.031) and lagging in central tumors at 23.08% versus 48.57% (*p* = 0.016). It is proven that transition from laparoscopic to robot assisted partial nephrectomy allowed us to apply more complex renal tumors to minimally invasive nephron-sparing techniques. Comparison of primary outcomes, which includes differences in RENAL score and TRIFECTA rate is presented in Table 2.

The TRIFECTA rate was higher in the RAPN group, but difference between two cohorts was not statistically significant. Differences in components of TRIFECTA, such as resection margin status, major postoperative complications, and postoperative renal function were insignificant too. The warm ischemia time was significantly shorter in RAPN with median of 17.5 min when compared to LPN with median of 22 min (*p* = 0.005).

***Postoperative outcomes.*** The differences in length of hospital stay, 30-day morbidity, postoperative complications, the necessity of second suture layer (double renorraphy), and operation time did not differ significantly between RAPN and LPN. Warm ischemia time was significantly shorter in RAPN when compared to LPN (Table 2 and Table 3).

Although lower WIT in the RAPN group suggested better postoperative renal function, the difference in eGFR was not statistically significant.

## 4. Discussion

In this retrospective comparative study, the authors presented a single-surgeon experience of transition from three-dimensional (3D) laparoscopic partial nephrectomy (LPN) to robot-assisted partial nephrectomy (RAPN) and compared the perioperative outcomes, complexity of cases (RANAL score), and achievement of the TRIFECTA criteria. We assessed 80 cases from the transition period.

Many studies compare conventional laparoscopy and robotic surgery in minimally invasive partial nephrectomy; therefore, most of them are non-randomized and retrospective. In the one of the latest reviews that follows PRISMA criteria, Guerrero et al. indicated that both techniques yield comparable oncological outcomes, length of stay (LOS), and positive margins. However, RAPN is advantageous in reducing warm ischemia time (WIT) and estimated blood loss, thereby supporting its use in more complex tumors [15,16,17].

While many investigations have explored the shift from open or standard laparoscopic approaches to robotic partial nephrectomy reports documenting a complete transition from three-dimensional laparoscopy to RAPN remain exceptionally scarce.

Three-dimensional laparoscopy provides depth perception, improving surgical precision and hand–eye coordination [18,19]. Since 3D laparoscopy imaging is closer to the view obtained in RAS, we hypothesized that the transition from three-dimensional laparoscopy to robotic platform should possibly be easier. It was well described that robotic systems made minimally invasive access more available for surgeons [20]. This is because, despite 3D vision, robotics added further enhancements, such as articulated instruments and tremor filtration [21]. This makes critical steps—particularly renal hilum control, a meticulous and precise tumor resection and intracorporeal suturing—more efficient and less dependent on advanced laparoscopic skills. Additionally, with a fourth robotic arm, the surgeon is less dependent on bedside assistance.

In meta-analysis from 2012, the authors did not find differences in tumor size between laparoscopic and robot-assisted nephron-sparing surgeries [22]. But in that paper, the RENAL score was not evaluated. RENAL score assessed the complexity of the tumor and it was proven that this scale can predict the risk of perioperative complications [23]. In our study, we revealed that patients in the RAPN group had statistically significant higher RENAL score compared to the LPN group. This confirms that robotic systems allow us to perform nephron-sparing surgery in more complex tumors, that would otherwise more likely be treated with radical nephrectomy in centers limited to laparoscopic techniques. Thus, robotic systems potentially reduce overtreatment with radical surgery by enabling excision in difficult locations.

Our analysis demonstrated that, despite significantly more complex tumors in the RAPN group, all perioperative outcomes were comparable or even superior in this cohort. Despite increased tumor complexity in the RAPN group, the rate of achieving TRIFECTA was higher, albeit not statistically significant. Moreover, the warm ischemia time (WIT) was significantly shorter in the RAPN group. This important parameter has the crucial impact of preserving renal function [24]. It was described that clamping of the renal artery should not exceed 20 or 25 min depending on the study [24,25]. This suggests enhanced capability for precise and efficient intracorporeal suturing, one of the most technically demanding aspects of NSS, which is greatly facilitated by the robotic platform. Similar results, favoring RAPN in warm ischemia time, were presented in few meta-analyses and reviews [22,26,27,28]. Importantly, in our series, as mentioned before, all procedures were performed using on-clamp technique. We are aware that a potentially off-clamp approach might have led to a better functional outcome, especially in a group of diabetic patients and those with impaired kidney function.

In the RAPN group, surgeons more often prefer to use only one layer of stiches (single renorrhaphy), while in the LPN group in most cases, a double layer was used. Single-renorrhaphy technique has a significant influence on warm ischemia time. It was proven that using a single layer of stitches shortens operative time, lowers estimated blood loss, and improves preservation of estimated glomerular filtration rate (eGFR) [23]. It can be caused by better suturing techniques when a robotic system is used. Better parenchymal preservation when single renorrhaphy is used and shorter warm ischemia time in the RAPN group is evidence of RAS being superior to laparoscopy in renal function preservation, which is the clue of NSS. It was proven in several meta-analyses that in the RAPN group, the decrease in eGFR was lower when compared to LPN [26,28,29].

In our study, the percentage of positive margines (R1) was comparable between both groups. Similar results were presented in another meta-analyses [26,28]. On the other site, in the biggest meta-analysis assessing 4 919 patients, the positive margin rate was significantly lower in the RAPN group [27].

In our analysis, no significant differences were observed between groups regarding hospital stay or perioperative complication rate. These findings are consistent with previous meta-analyses [26,29], some of which even reported lower complication rates for RAPN [27,28].

There were also other advantages of RAPN over LPN described in the literature. Authors proved that in robot-assisted partial nephrectomies, there was lower conversion rate to open surgery or to the radical nephrectomy [27,28,29]. Furthermore, as highlighted by Andras et al. [30], advances in minimally invasive techniques—particularly three-dimensional laparoscopy—have considerably expanded the feasibility of complex renal procedures and helped maintain high surgical standards in experienced centers. Our results build upon this concept, showing that the transition to robotic-assisted surgery represents a natural continuation of this progress, offering additional precision and ergonomics that further enhance the safety and applicability of nephron-sparing surgery in anatomically demanding cases. Thus, over the last decade, the transition from open to robotic surgery led to the reduction in cases of nephrectomies and the increase in nephron-sparing approaches even in the most complex tumors.

## 5. Limitations

Several limitations should be acknowledged. This was a non-randomized, retrospective, single-center study with a relatively small sample size. However, we mitigated selection bias through rigorous propensity score weighting and multiple imputation of missing data, preserving statistical power and improving group comparability. The sample included all consecutive cases operated by a single surgeon, which reduces variability but may limit generalizability. Clamping of renal pedicle might be considered as another drawback of our study and we are aware that a potentially off-clamp approach might have led to a better functional outcome, especially in a group of diabetic patients and those with impaired kidney function.

## 6. Conclusions

In conclusion, the transition from three-dimensional laparoscopic partial nephrectomy to robot-assisted partial nephrectomy performed by experienced surgeon proved to be safe and feasible, with no compromise in perioperative or oncological outcomes. Importantly, this study does not compare the robotic platform with conventional two-dimensional laparoscopy, but rather with an advanced 3D laparoscopic technique that already provides depth perception and enhanced spatial orientation. Therefore, our results demonstrate that even when benchmarked against the most optimized form of laparoscopy, robotic assistance offers comparable or superior outcomes, particularly in more complex renal tumors. The robotic system further expands the applicability of nephron-sparing surgery by enabling safe resection of anatomically challenging lesions that might otherwise require radical nephrectomy. These findings emphasize that for surgeons experienced in 3D laparoscopy, the complete transition to robotic partial nephrectomy can be achieved smoothly, maintaining surgical quality and broadening the indications for minimally invasive nephron-sparing surgery. These observations may serve as valuable insights for emerging robotic urology teams and provide a realistic basis for patient counseling during the transition from laparoscopic to robotic PN.

## Figures and Tables

**Table 1:** Patient characteristics.

Characteristic	LPN (*N* = 36)	RAPN (*N* = 44)	Overall Sample (*N* = 80)	*p*-Value
**Demographics**				
Sex, *n* (%)				0.440
Female	17.0 (47.2%)	17.0 (38.6%)	34.0 (42.5%)	
Male	19.0 (52.7%)	27.0 (61.3%)	46.0 (57.5%)	
Age, median (IQR), years	66.0 (62.5, 70.0)	64.0 (47.7, 72.2)	65.0 (57.0, 70.2)	0.165
BMI, median (IQR), kg/m^2^	27.8 (26.0, 31.0)	29.0 (25.8, 32.6)	28.0 (26.0, 32.4)	0.783
**Preoperative Assessment**				
ASA Score, *n* (%)				
II	9.0 (25.0%)	2.0 (4.7%)	11.0 (13.9%)	**0.010**
III	27.0 (75.0%)	40.0 (93.0%)	67.0 (84.8%)	0.071
IV	0.0 (0.0%)	1.0 (2.3%)	1.0 (1.3%)	1.000
Hypertension, *n* (%)	20.0 (55.6%)	24.0 (54.6%)	44.0 (55.0%)	0.928
Diabetes, *n* (%)	8.0 (22.2%)	10.0 (22.7%)	18.0 (22.5%)	0.957
Obesity, *n* (%)	12.0 (33.3%)	20.0 (45.5%)	32.0 (40.0%)	0.271
Oral Anticoagulation, *n* (%)	3.0 (8.3%)	4.0 (9.1%)	7.0 (8.8%)	1.000
Previous surgery, *n* (%)	14.0 (38.9%)	23.0 (52.3%)	37.0 (46.3%)	0.232
Creatinine Baseline, median (IQR)	0.9 (0.8, 1.1)	0.9 (0.8, 1.1)	0.9 (0.8, 1.1)	0.862

**Table 2:** RENAL score and TRIFECTA.

Characteristic	LPN (*N* = 36)	RAPN (*N* = 44)	Overall Sample (*N* = 80)	*p*-Value
**RENAL nephrometry**				
RENAL Score, median (IQR)	6.0 (4.0, 7.0) (*N* = 35)	8.0 (6.0, 9.0) (*N* = 39)	7.0 (5.0, 8.0) (*N* = 74)	**0.001**
RENAL Complexity Category, *n* (%)			*N* = 74	**0.001**
Low	23.0 (65.7%)	11.0 (28.2%)	34.0 (46.0%)	**0.001**
Medium	12.0 (34.3%)	24.0 (61.6%)	36.0 (48.7%)	**0.073**
High	0.0 (0.0%)	4.0 (10.3%)	4.0 (5.4%)	**0.123**
Tumor Location (RENAL), *n* (%)			*N* = 74	**0.020**
Anterior	11.0 (31.4%)	11.0 (28.2%)	22.0 (29.7%)	0.622
Central	17.0 (48.6%)	9.0 (23.1%)	26.0 (35.1%)	**0.016**
Posterior	7.0 (20.0%)	19.0 (48.7%)	26.0 (35.1%)	**0.031**
**TRIFECTA**				
TRIFECTA, *n* (%)	15.0 (42.9%)	26.0 (60.5%)	41.0 (52.7%) (*N* = 78)	0.121
Resection Margin (R Status), *n* (%)			*N* = 75	1.000
R0	27.0 (87.1%)	34.0 (89.5%)	67.0 (89.3%)	
R1	4.0 (12.9%)	4.0 (10.5%)	8.0 (11.6%)	
Ischemia Time, median (IQR), min	22.0 (16.0, 24.0)	17.5 (14.9, 23.0)	18.0 (15.0, 23.0)	**0.005**
Major Postoperative Complications (CD III-V), *n* (%)	3.0 (8.3%)	2.0 (4.6%)	5.0 (6.3%)	0.653
eGFR Postoperative (mL/min/1.73 m^2^), *n* (%)	*N* = 72			0.326
60 or Less	10.0 (32.3%)	9.0 (22.0%)	19.0 (26.4%)	
Above 60	21.0 (67.7%)	32 (78.1%)	53.0 (73.6%)	

**Table 3:** Postoperative outcomes.

Characteristic	LPN (*N* = 36)	RAPN (*N* = 44)	Overall Sample (*N* = 80)	*p*-Value
**Tumor characteristics** Histology, *n* (%)				0.765
Clear Cell RCC	24 (66.7%)	34 (77.3%)	58 (72.5%)	0.384
Chromophobe RCC	3 (8.3%)	2 (4.5%)	5 (6.2%)	0.653
Oncocytoma	2 (5.6%)	3 (6.8%)	5 (6.2%)	1.000
Papillary RCC	3 (8.3%)	3 (6.8%)	6 (7.5%)	1.000
Other tumors	3 (8.3%)	2 (4.5%)	5 (6.2%)	0.653
**Intraoperative Details**				
Renorrhaphy, *n* (%)				0.100
Double	23 (63.9%)	20 (45.4%)	43 (53.7%)	
Single	13 (36.1%)	24 (54.5%)	37 (46.2%)	
Surgery Time, median (IQR), min	140.0 (120.0, 165.0)	145.0 (130.0, 180.0)	145.0 (125.0, 170.0)	0.410
**Postoperative Outcomes**				
LOS, median (IQR), days	5.0 (4.0, 6.0)	5.0(4.0, 5.0)	5.0 (4.0, 5.0)	0.079
30-day Morbidity, *n* (%)	7 (19.4%)	6 (13.6%)	13 (16.2%)	0.484

**Table 4:** Propensity score weighting.

Variable	Effect Measure	Estimate (95% CI)	*p*-Value
*Outcome variables*			
TRIFECTA achievement	OR	1.7 (0.5, 6.0)	0.374
Length of stay (LOS), days	β	−1.7 (−4.3, 0.9)	0.193
Minor postoperative complications (CD I-II)	OR	1.5 (0.2, 9.4)	0.671
Major postoperative complications (CD III-V)	OR	0.2 (0.0, 1.6)	0.113
Any postoperative complications (CD I-V)	OR	0.6 (0.1, 3.4)	0.550
Postoperative creatinine, mg/dL	β	−0.0 (−0.2, 0.2)	0.771
Postoperative eGFR (>60 mL/min/1.73 m^2^)	OR	1.4 (0.3, 6.2)	0.668
*Intraoperative details*			
Surgery time, minutes	β	5.5 (−21.0, 32.1)	0.679
Ischemia time, minutes	β	−4.2 (−7.2, −1.0)	**0.008**

*Notes:* *OR*: Odds ratio; *β*: Regression coefficient; 95% *CI*: 95% confidence interval; *p*-value: Statistical significance (*p* < 0.05 indicates significance).

## Data Availability

Data supporting the findings are available from corresponding author upon reasonable request.

## Abbreviations

ASA	American Society of Anesthesiologists
BMI	Body Mass Index
CD	Clavien–Dindo (classification of surgical complications)
CI	Confidence Interval
eGFR	Estimated Glomerular Filtration Rate
IC	Informed Consent
IQR	Interquartile Range
LPN	Laparoscopic Partial Nephrectomy
LOS	Length of Stay
MIS	Minimally Invasive Surgery
NSS	Nephron-Sparing Surgery
OR	Odds Ratio
PN	Partial Nephrectomy
PSW	Propensity Score Weighting
RAPN	Robot-Assisted Partial Nephrectomy
RCC	Renal Cell Carcinoma
RENAL	Radius, Exophytic/Endophytic, Nearness to collecting system, Anterior/Posterior, and Location relative to polar lines (Nephrometry Score)
RN	Radical Nephrectomy
R0/R1	Negative/Positive Surgical Margin
SMD	Standardized Mean Difference
TRIFECTA	Composite outcome including negative surgical margins, no major complications, and preserved renal function (WIT < 25 min, >90% eGFR preservation)
WIT	Warm Ischemia Time

## References

[1] Ferlay J., Colombet M., Soerjomataram I., Dyba T., Randi G., Bettio M., Gavin A., Visser O., Bray F. (2018). Cancer incidence and mortality patterns in Europe: Estimates for 40 countries and 25 major cancers in 2018. Eur. J. Cancer.

[2] Bray F., Laversanne M., Sung H., Ferlay J., Siegel R.L., Soerjomataram I., Jemal A. (2024). Global cancer statistics 2022: GLOBOCAN estimates of incidence and mortality worldwide for 36 cancers in 185 countries. CA Cancer J. Clin..

[3] O’Connor E., Timm B., Lawrentschuk N., Ischia J. (2020). Open partial nephrectomy: Current review. Transl. Androl. Urol..

[4] Katsimperis S., Tzelves L., Bellos T., Manolitsis I., Mourmouris P., Kostakopoulos N., Pyrgidis N., Somani B., Papatsoris A., Skolarikos A. (2024). The use of indocyanine green in partial nephrectomy: A systematic review. Cent. Eur. J. Urol..

[5] Ren K., Wu F., Wu H., Ning H., Lyu J. (2024). Partial versus radical nephrectomy for T1b renal cell carcinoma: A comparison of efficacy and prognostic factors based on the Surveillance, Epidemiology, and End Results database. Curr. Urol..

[6] Bex A., Ghanem Y.A., Albiges L., Bonn S., Campi R., Capitanio U., Dabestani S., Hora M., Klatte T., Kuusk T. (2025). European Association of Urology Guidelines on Renal Cell Carcinoma: The 2025 Update. Eur. Urol..

[7] Van Poppel H., Da Pozzo L., Albrecht W., Matveev V., Bono A., Borkowski A., Colombel M., Klotz L., Skinner E., Keane T. (2011). A prospective, randomised EORTC intergroup phase 3 study comparing the oncologic outcome of elective nephron-sparing surgery and radical nephrectomy for low-stage renal cell carcinoma. Eur. Urol..

[8] Winfield H.N., Donovan J.F., Lund G.O., Kreder K.J., Stanley K.E., Brown B.P., Loening S.A., Clayman R.V. (1995). Laparoscopic partial nephrectomy: Initial experience and comparison to the open surgical approach. J. Urol..

[9] Sinha R.Y., Raje S.R., Rao G.A. (2017). Three-dimensional laparoscopy: Principles and practice. J. Minim. Access Surg..

[10] Schmidt M.W., Fan C., Köppinger K.F., Schmidt L.P., Brechter A., Limen E.F., Vey J.A., Metz M., Müller-Stich B.P., Nickel F. (2024). Laparoscopic but not open surgical skills can be transferred to robot-assisted surgery: A systematic review and meta-analysis. World J. Surg..

[11] Karavitakis M., Grivas N., Zabaftis C., Nikitakis F., Tsela S., Leotsakos I., Katafigiotis I., Mitropoulos D. (2025). The influence of 3D technology integration on laparoscopic partial nephrectomy practice and surgical outcomes. Curr. Oncol..

[12] Shiroki R., Fukami N., Fukaya K., Kusaka M., Natsume T., Ichihara T., Toyama H. (2016). Robot-assisted partial nephrectomy: Superiority over laparoscopic partial nephrectomy. Int. J. Urol..

[13] Fiorello N., Di Benedetto A., Summonti D., Mogorovich A., Sepich C.A. (2021). Learning curve in robot-assisted partial nephrectomy: Comparison between an expert surgeon and a team in training in single-center experiences. Cent. Eur. J. Urol..

[14] Mihai I., Dura H., Teodoru C.A., Todor S.B., Ichim C., Grigore N., Mohor C.I., Mihetiu A., Oprinca G., Bacalbasa N. (2024). Intraoperative ultrasound: Bridging the gap between laparoscopy and surgical precision during 3D laparoscopic partial nephrectomies. Diagnostics.

[15] Ruiz Guerrero E., Claro A.V.O., Ledo Cepero M.J., Soto Delgado M., Álvarez-Ossorio Fernández J.L. (2023). Robotic versus laparoscopic partial nephrectomy in the new era: Systematic review. Cancers.

[16] Lavery H.J., Small A.C., Samadi D.B., Palese M.A. (2011). Transition from laparoscopic to robotic partial nephrectomy: The learning curve for an experienced laparoscopic surgeon. JSLS.

[17] Kumar S., Nayak B. (2024). Transition from open and laparoscopic to robotic partial nephrectomy: Learning curve and outcomes. Cureus.

[18] Nguyen D.H., Nguyen B.H., Van Nong H., Tran T.H. (2019). Three-dimensional laparoscopy in urology: Initial experience after 100 cases. Asian J. Surg..

[19] Izquierdo L., Peri L., García-Cruz E., Musquera M., Ciudin A., Pérez M., Alcaraz A. (2012). 3D advances in laparoscopic vision. Eur. Urol. Rev..

[20] Patel M.N., Bhandari M., Menon M., Rogers C.G. (2009). Robotic-assisted partial nephrectomy. BJU Int..

[21] Rogers C.G., Singh A., Blatt A.M., Linehan W.M., Pinto P.A. (2008). Robotic partial nephrectomy for complex renal tumors: Surgical technique. Eur. Urol..

[22] Aboumarzouk O.M., Stein R.J., Eyraud R., Haber G.P., Chlosta P.L., Somani B.K., Kaouk J.H. (2012). Robotic versus laparoscopic partial nephrectomy: A systematic review and meta-analysis. Eur. Urol..

[23] Hew M.N., Baseskioglu B., Barwari K., Axwijk P.H., Can C., Horenblas S., Bex A., de la Rosette J.J.M.C.H., Laguna Pes M.P. (2011). Critical appraisal of the PADUA classification and assessment of the R.E.N.A.L. nephrometry score in patients undergoing partial nephrectomy. J. Urol..

[24] Thompson R.H., Lane B.R., Lohse C.M., Leibovich B.C., Fergany A., Frank I., Gill I.S., Blute M.L., Steven C. (2010). Campbell Every minute counts when the renal hilum is clamped during partial nephrectomy. Eur. Urol..

[25] Becker F., Van Poppel H., Hakenberg O.W., Stief C., Gill I., Guazzoni G., Montorsi F., Russo P., Stöckle M. (2009). Assessing the impact of ischaemia time during partial nephrectomy. Eur. Urol..

[26] Choi J.E., You J.H., Kim D.K., Rha K.H., Lee S.H. (2015). Comparison of perioperative outcomes between robotic and laparoscopic partial nephrectomy: A systematic review and meta-analysis. Eur. Urol..

[27] Leow J.J., Heah N.H., Chang S.L., Chong Y.L., Png K.S. (2016). Outcomes of robotic versus laparoscopic partial nephrectomy: An updated meta-analysis of 4919 patients. J. Urol..

[28] Jiang Y.L., Yu D.D., Xu Y., Zhang M.H., Peng F.S., Li P. (2023). Comparison of perioperative outcomes of robotic vs. laparoscopic partial nephrectomy for renal tumors with a RENAL nephrometry score ≥7: A meta-analysis. Front. Surg..

[29] Buckland B., Tree K., Best O., Heijkoop B., Senanayake T., Handmer M. (2024). Robotic versus laparoscopic partial nephrectomy: A systematic review and meta-analysis of randomised trials. Surg. Technol. Int..

[30] Andras I., Territo A., Telecan T., Medan P., Perciuleac I., Berindean A., Stanca D.V., Buzoianu M., Coman I., Crisan N. (2021). Role of the laparoscopic approach for complex urologic surgery in the era of robotics. J. Clin. Med..

