# Correction: Kania et al. Evaluating the Transition from 3D Laparoscopy to Robotic Partial Nephrectomy: Trifecta Achievement and Nephrometry Score Differences. *Cancers* 2025, *17*, 3976

**DOI:** 10.3390/cancers18203314

**Published:** 2026-10-14

**Authors:** Piotr Kania, Paweł Marczuk, Jakub Biedrzycki, Markijan Kubis, Szymon Kania, Kajetan Juszczak, Maciej Salagierski

**Affiliations:** 1 Department of Urology and Urological Oncology, St. John Paul II Masovian Regional Hospital, Ks. Jozefa Poniatowskiego 26, 08-110 Siedlce, Poland; 2 Faculty of Medicine, University of Siedlce, 08-110 Siedlce, Poland; 3 Urology Department, Collegium Medicum, University of Zielona Góra, 65-417 Zielona Góra, Poland; 4 Medical University of Lublin, 20-059 Lublin, Poland; 5 Department of Urology and Andrology, Collegium Medicum, Nicolaus Copernicus University, 85-067 Bydgoszcz, Poland

In the published publication [1], there was an error regarding the affiliation for Szymon Kania. In addition to the affiliation ^1^ Department of Urology and Urological Oncology, St. John Paul II Masovian Regional Hospital, Ks. Jozefa Poniatowskiego 26, 08-110 Siedlce, Poland, the update should include the affiliation ^4^ Medical University of Lublin, 20-059 Lublin, Poland. 

The authors state that the scientific conclusions are unaffected. This correction was approved by the Academic Editor. The original publication has also been updated.
